# Arab male physicians’ perceptions about their own smoking behaviors: a qualitative study

**DOI:** 10.1186/s13584-024-00602-2

**Published:** 2024-04-02

**Authors:** Samira Obeid, Nasra Idilbi, Abed Agbarya, Hanna Admi

**Affiliations:** 1 Nursing Department, The Max Stern Yezreel Valley Academic College, D.N. Emek Yezreel, 1930600 Israel; 2 Ministry of Health, Northern Region, Nof Hagalil, 1710602 Israel; 3 Galilee Medical Center, Nahariya, 22100 Israel; 4 Bnai Zion Medical Center, Sderot Eliyahu Golomb 47, Haifa, Israel

**Keywords:** Arabs, Cessation, Israel, Physicians, Smoking

## Abstract

**Background:**

Smoking remains the leading preventable cause of disease, disability, and death worldwide. Although physicians have high levels of health literacy with awareness of the consequences of smoking and their essential role in smoking cessation of patients, some physicians continue to smoke. Rates of smoking among Arab male physicians are high. This study aimed to gain insights into Arab male physician’s perceptions of their own smoking behaviors and their professional role in health promotion.

**Methods:**

Using purposive sampling, we recruited 25 Arab male physicians working in hospital and community clinic settings who currently smoke. Semi-structured, hour-long, interviews were held during January—June 2022. We then performed a thematic analysis of the interview data.

**Results:**

The analysis revealed three categories, two sub-categories, and 15 emerging themes. The category ‘Antecedents: prior to becoming a physician’ revealed the themes: smoking experience during adolescence; social and ethnic culture; stress during medical studies; and on & off periods of quitting smoking. The category ‘Physicians’ perception of smoking’ was sorted into two sub-categories: (1) Personal aspects, including the themes ‘relaxation from stress’, ‘self-compensation’, ‘addiction’, and ‘enjoyable experience’, and (2) Professional aspects, including the themes ‘lack of knowledge about cessation’, ‘inadequate workplace support’, ‘motivation to consult patients’, and ‘awareness of their role as primary care physicians’. The category’Impacts’ revealed the themes ‘personal health and well-being’, ‘professional competence’, and ‘professional image in public’.

**Conclusions:**

This study provides an in-depth understanding of the personal, socio-cultural, and professional aspects of the phenomenon of Arab male smoking physicians from their perspective. Based on this information, we recommend developing programs that support and empower all physicians to cope better with their personal and professional stress as well as instituting programs that will provide all physicians with specific knowledge and skills related to smoking cessation. These programs should improve the ability of physicians to serve as positive role models for their patients for preventing and ceasing smoking, thus enhancing the image of the medical profession and, most importantly, improving the health of the public.

## Background

Smoking severely affects the health of individuals and those exposed to secondhand smoke. Smoking, which continues to be a significant public health issue worldwide, kills more than eight million people annually. The World Health Organization emphasizes the role of physicians in counseling their patients in favor of smoking cessation [1]. Research shows that even a small question from the physician to the patient about their smoking status could be effective in causing a patient to quit smoking [2].

A Cochrane review on the role of physician’s advice asserted that when a physician counsels a patient to quit smoking, it increases patient quitting rates. Additionally, when advised more intensely, smoking cessation rates increased up to 65% [3]. Many interventions have been described to encourage physicians to counsel patients to quit smoking [4–8]; however, counseling rates remain low. Family physicians should consider the stage at which smokers are. For those who are in the "pre-contemplation" stage of quitting smoking, a discussion with their physician can encourage some to begin to contemplate cessation [9, 10]. Furthermore, previous studies have found that physicians who smoke are less likely to manage smoking cessation among their patients [11, 12].

Even though physicians have knowledge of the consequences of smoking and an essential role in encouraging patients to quit smoking, some physicians continue to smoke. A study on smoking prevalence among physicians was conducted in 2021 [13], which described the rates of smoking by specialty. The results showed that among 246 studies and 497,081 physicians, smoking prevalence was 21% (95 CL 20% to 23%). The highest prevalence was amongst medical students and family practitioners (25% and 24%, respectively), and the lowest amongst anesthesiologists, radiologists, and pediatricians (11%, 9%, and 8%, respectively).

The smoking prevalence of physicians varies between developed and developing countries [14]. Physicians from Central/Eastern Europe have the highest prevalence (37%), followed by those from Africa (29%), Central and South America (25%), and Asia (17.5%). The rates of smoking among physicians in the US were the lowest in 2010–2011, 1.95% [15]. The smoking prevalence of Israeli Jewish physicians varies between 8% [16] and 16.7% [17].

Many factors influence physician smoking, including gender (men smoke more than women) [13], workload and stress [18], depressive disorders [19], nightshifts [20], and other factors that disrupt the circadian rhythm and can increase smoking [21]. Family history of smoking and physician marital status (being unmarried) also influence smoking behaviors [22]. Married individuals derive advantages from accessing spousal support and specialized coping resources inherent to the marital bond [23]. Conversely, those without such support are at a higher risk of encountering social isolation or disconnection, a recognized key factor contributing to adverse health behaviors [24]. Age has been found to be a factor of importance, since physicians who started smoking at a younger age have developed heavier addictions to tobacco [25]. In addition, an analysis of the academic literature on the factors related to smoking by Israeli Jewish physicians emphasized two contributing factors: the physician type of specialization (being surgeons) [16] and stress [17].

The Theory of Planned Behavior can serve to understand the complexity of factors related to healthy and unhealthy behaviors, such as smoking [26]. According to the theory, there are background factors including characteristics of individuals (e.g., personality factors, experiences, and attitudes); social factors (e.g., ethnicity and culture); and information factors (e.g., knowledge and media). Interventions can influence behavioral, normative, and control beliefs. Such beliefs are related to whether the adoption of specific behaviors can be effective in producing positive health outcomes, and whether individuals can adopt these behaviors successfully (control beliefs). These beliefs can influence attitudes related to the adoption of specific behaviors, intentions to engage in these behaviors, and ultimately promote behavior changes [26].

### Israeli Arab physicians

The Arab community is an ethnic minority in Israel. Arabs represents 21% of the population and have a relatively lower socioeconomic status as compared to the Jewish population. Most Arabs live in peripheral areas and suffer poorer health outcomes [27]. Smoking rates among Arab Israelis are higher than in Jewish populations for all ages. A 2020 Ministry of Health report documented that overall, 20.1% of Israeli citizens smoked. The overall smoking rates of Arabs (24.4%) were higher than for Jews (19.1%). This reflects the fact that twice as many Arab men (44%) reported smoking as Jewish men (22%), while in contrast, the smoking rate for Arab Israeli women was lower than that of Jewish women (10.2% vs. 15.8%) [28]. By 2021, 21% of the 32,000 physicians in Israel were Arab, and of the 6720 Arab physicians, 57% were male [29].

## Methods

This study aimed to understand the perceptions of Arab Israeli male physicians about factors related to their smoking and about their role as physicians in promoting smoking cessation. We performed a qualitative study of Arab Israeli male physicians who smoke with thematic analysis of the data collected in interviews. To guide and report the research, we used COREQ (the Consolidated criteria for Reporting Qualitative studies [30]. It provides a checklist of the items that should be included in reports of qualitative research.

### Participant selection

We used convenience and snowball sampling to recruit 25 Arab male physicians from the central and northern regions of Israel. The researchers work in various institutions and across multiple regions in the country. We recruited the initial participants through the professional networks of the researchers. Then each participant was asked to give the interviewer names of smoking physicians who might agree to be interviewed from their friends and acquaintances’ networks. Inclusion criteria included male physicians of all specialty areas, who were current smokers and over the age of 25 years, the approximate age of medical school graduates in Israel. We approached 200 smoking male physicians of whom 25 (12.5%) agreed to participate by providing information in an approximately one-hour long semi-structured interview. We decided to interview only men because Arab women smoke much less than Arab men. Therefore, it would have been particularly challenging to find female Arab physicians who smoked and agreed to be interviewed.

### Data collection

Skilled research assistants conducted semi-structured face-to-face interviews using interview guides. The interview guide (Appendix) included questions like: “What do you think about smoking among physicians?”, “What do you think are the reasons for Arab physicians smoking?”, and “What do you think are your patients' attitudes towards your smoking?”.

Each interview lasted about an hour. Interviews were conducted in Arabic, recorded, transcribed, and translated to English.

### Data analysis

We performed an analysis I based on the constant comparative method of qualitative data analysis [30]. The inductive analytic process included coding the raw data, classifying the codes into categories, and identifying emerging themes from the data and field notes. The final stage integrated the categories into a cohesive theory. All members of the research team engaged in comprehensive discussions about the stages of analysis and reached a consensus regarding the initial categorization and insights.

## Results

### Participants

We interviewed the 25 Arab male physician participants between January-June, 2022. Their demographic information is presented in Table 1.

**Table 1:** Demographic Information (*N* = 25)

**Variable**	**Participants %(N)** ^**a**^
Gender, male	100 (25)
Age, years – mean (std)	36 (10.28)
**Work setting**	
Hospital	60 (14)
Community	20 (7)
Hospital and community	20 (4)
**Field of expertise**	
Family Medicine	20 (5)
Rehabilitation	4 (1)
Internal Medicine	28 (7)
Surgery	16 (4)
Diabetes and endocrinology	12 (3)
Geriatrics	8 (2)
Neurology	8 (2)
Hospice	4 (1)
**Marital status**	
Married	56 (14)
Single	40 (10)
Engaged	4 (1)
**Smoking Type**	
Cigarettes	72 (18)
Nargila	28 (7)
E-cigarettes	0 (0)
**Age of smoking initiation**	
16–27 years	64 (16)
≥28 years	36(9)
Smoking duration, years – mean (std)	8 (5.74)
**Smoking initiation time**	
Before starting medical school	56 (14)
During medical school studies	44 (11)
**Attempted to quit smoking**	48 (12)

^a^ Unless otherwise stated

### Findings

We sorted all the data into three categories and two sub-categories. Fifteen themes emerged.

**Fig. 1:**
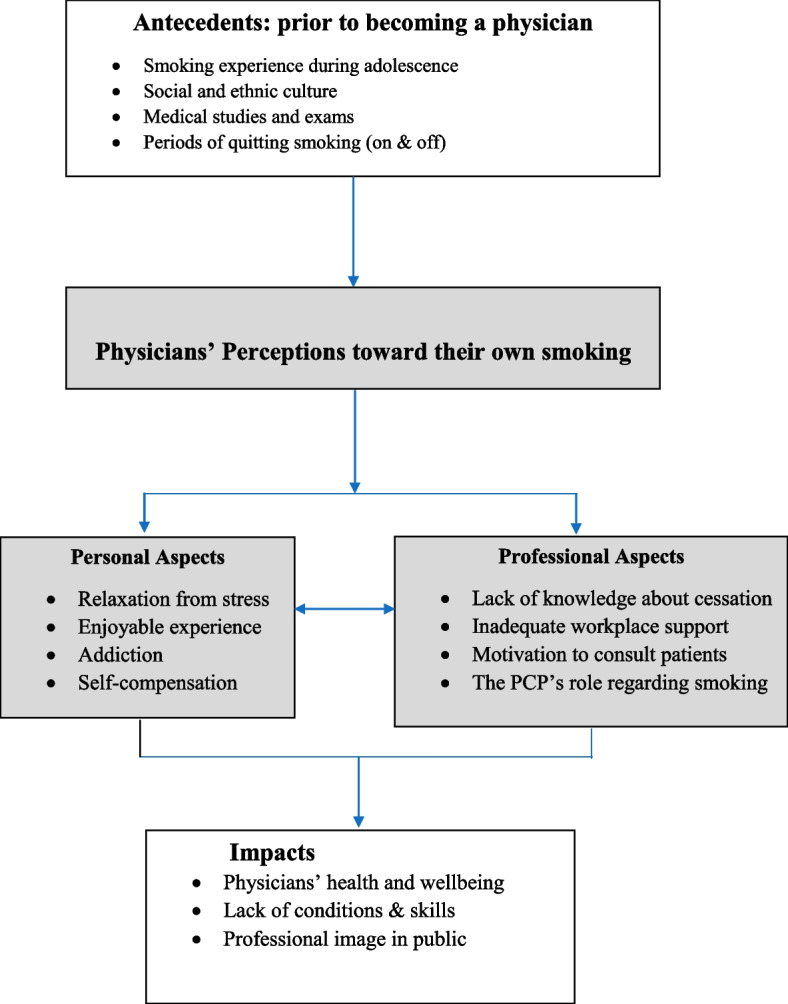
Arab smoking physicians: a schematic model of categories, sub-categories, themes, and relationships among them

They are summarized in Fig. 1.

For the category “Antecedents: smoking-related factors prior to becoming a physician”, we identified the following themes:

#### Smoking experience during adolescence

Most (64%) of the physicians started smoking at younger ages (16–27 years) during high school and university. They talked about their earlier smoking experience. For example, a 36-year-old internal medicine physician said:

”Most people start to smoke at a young age, while in [high] school… When you learn and see how much [cigarettes] cause damage, you are already in a situation in which it will not be easy to stop.”

#### Social and ethnic culture factors

A 29-year-old physician working in the community said:

"I started smoking as a result of peer pressure from my Arab friends. When we had social gatherings, they would give me [cigarettes], and at a certain point, it was not comfortable for me [to keep taking cigarettes from them]. So I started to buy packs of cigarettes."

Moreover, participants reported that smoking was prevalent amongst their peer group of Arab physicians at their place of work. One physician, a 30-year-old rehabilitation physician, noted:

"Of course, the percentage of smokers is more amongst [Arab] physicians; I know physicians that, before they begin a shift that starts at 3:00 or 4:00, go to the supermarket and buy two packs of cigarettes or [even] four so they will not run out. They are my friends, Arab [smoking physicians]. I have never seen a Jew[ish] [physician] smoke."

These physicians’ reports illustrate the frequent smoking of Arab male physicians and how this can affect the likelihood of a physician smoking in the workplace.

#### Medical studies and exams

A salient theme emerged throughout interviews whereby participants reported that medical school and the state medical licensing exam were periods of smoking initiation or intensification. A rehabilitation physician working in a hospital and the community remarked:

"When I was young and started [smoking] during the examination period, I understood that this was not so good, and when I came back here, there was the state test. That raised the emotional stressors, and I started smoking heavily."

#### Periods of quitting smoking

The participants discussed the difficulty of quitting smoking and mentioned that they had experienced attempts to quit and then returned to smoking for various reasons.

### Physicians’ perceptions toward their own smoking

We sorted the category “Physicians’ perceptions toward smoking” into two sub-categories: Personal aspects (2.1) and Professional aspects (2.2).

#### Personal aspects

Personal motivations for smoking included three further sub-categories:

##### Relaxation from stress

Participants identified many stressors affecting their decision to begin smoking. They noted that general life stressors (work, family, finances, and “other”) were related to their initiating and continuing smoking and felt that their smoking helped them in coping with these stressors.

“Shift work, stress from work, being on call. With the hard shifts, despite the stress, you have to make the appropriate decisions for the patient. Therefore, you feel stressed and need to escape to smoke, calm down, and return to work.”

Because of the difficulties they experience on a daily basis, the physicians reported finding comfort in smoking, as expressed in the above example.

##### Addiction

While for some physicians smoking was a behavior they could maintain without seeming to develop an addiction, (i.e., they could smoke sporadically), for others, smoking became an addiction. They describe how in Arab society, smoking behaviors learned in adolescence can develop into powerful addictions. Even when physicians fully understand the impacts of smoking, they find these addictions very difficult to break.

Furthermore, in most cases, the learned behavior of smoking in a social context with a peer group led to more severe addictions among the physicians.

##### Enjoyable experiences

Other physicians reported that smoking was a recreational activity that they specifically enjoyed while off-shift. One physician stated that smoking a cigarette is a leisure activity that makes him feel good. He said:

"I do not smoke because I am stressed, the opposite, I smoke only when I am relaxed with my family and friends.

#### Professional aspects

We identified four themes related to professional aspects of smoking:

##### Lack of knowledge about cessation

Physicians participating in this study expressed limited knowledge regarding pathways to smoking cessation and treatment options. For example, when asked what methods they knew about quitting smoking, a 29-year-old geriatrics physician responded:

“There is gum and all kinds of pills…”

Further, when asked the same question, a 30-year-old neurology physician responded: “I do not know, but I heard there are lectures and stickers and gum that can help..."

On the other hand, physicians also reported an openness to, and interest in, learning more methods for smoking cessation.

##### Inadequate workplace support

The participants indicated that their workplaces do not support them in the smoking cessation process. They provided several suggestions about ways their workplaces could assist them.

For example, a 29-year-old community physician working in a hospital setting noted:

“…In my opinion, there need to be smoking cessation workshops for physicians. If they are at the hospital, I will sign up because it is accessible and easy to participate. I think limiting smoking in public places does not help. If you isolate them [smokers] and make them feel like outcasts - that will not help them stop smoking. It is not a matter of knowledge but rather psychology - how much you can influence them to leave the addiction. The physicians know the risks of smoking, so it is not about raising knowledge.”

Some physicians suggested gyms or sports teams that they would be able to attend during working hours as a means of coping with the professional stressors that they experience.

##### Motivation to counsel patients

Physicians experience conflict in counseling patients to quit smoking when they have not succeeded in doing so themselves. Some find it stressful to be in that situation, while others go so far as to reject the premise that they should be a role model for their patients.

However, one 30-year-old internal medicine physician working in a hospital setting noted:

"The truth is that it [the encounter with patients about smoking] influences me a lot, not just them seeing me smoking, but the smell when I go to the patient, and I tell them to stop smoking, and I smell like a smoker. In general, at work, I try not to smoke.”

Despite this, all the physicians in this study did report that they counseled patients to reduce and quit smoking.

Apparent in the responses from the participants is that this internal conflict and dialogue has a daily impact on the level of tension the physicians' experience regarding their smoking habits. It causes some level of reflection in each patient encounter.

##### The Primary Care Physician’s (PCP) role regarding smoking

A salient theme emerged in the division of labor between hospital-based specialist physicians and family physicians. Specialists understood themselves as responsible for explaining continued smoking risks to their patients and referring them to their family physicians for follow-up. The family physician was identified as the relevant figure to encourage smoking cessation based on their relationship with the patient. A 30-year-old hospital-based internal medicine physician explains:

"…the patient always trusts the physician - [family physician]. There is a connection between them - beyond the physician-patient relationship in a hospital…a patient comes for 2 or 3 days, is treated, and leaves. ….. So, when I suggest to the patient [to stop smoking, let us say I have known him for 2 days. They will not relate to that like they would to their family physician, who knows them for 10 or 15 years.”

Physicians in this study reported that upon discharge, they referred patients to their family physician for further guidance about smoking cessation.

We found three themes in the last category “Impacts:”

### Impacts

#### Physicians’ health and well-being

Participants were highly aware of the risks of smoking and being smokers. However, despite this, some of them continued smoking.

A 27-year-old community physician reflected on his smoking behaviors:

“Look, I know it is dangerous, but I say - I am still young, so [smoking] will not do anything to me right now.”

#### Inadequate conditions and training

Hospital-based physicians expressed a lack of time and resources to counsel patients to quit smoking. These physicians felt they did not have enough time or knowledge to provide the level of counseling a patient might need to quit smoking. A 30-year-old internal medicine physician remarked:

"... There is not enough time to speak with the patient. For instance, if there is a patient after a heart attack, you tell [the patient] that [they] need to take care, etc., and say a few words to them. I do not have the tools or ways to help them stop smoking. Because of this, we refer them to their clinics [family physicians] who can help them."

Overall, physicians expressed openness to, and interest in, more training to gain more skills to counsel patients toward smoking cessation.

#### Professional image in public

Physicians understand that they need to serve as role models in the field of health promotion. Therefore, they refrain from smoking in the presence of others, both to avoid harming the public and because they realize that it negatively impacts their professional image.

A 29-year-old geriatrics physician working in a community setting reflected:

"I try to smoke in places where there are [other] smokers so that I will not harm someone [not smoking]; I look for places where smoking is permitted and smoke.”

The physicians also shared the conflict between the public's expectations of them to serve as role models for health-promoting behavior and their smoking habits. A 30-year-old internal medicine physician who works in a hospital reflected,

"The truth is, I do not like the feeling [of being seen smoking at the hospital], I try and avoid patients and people who relate to me like a physician. When I want to smoke, I go to the smoking corner by the M.R.I., where usually most [of the smokers] are staff and not patients and their families.”

## Discussion

Smoking causes countless preventable morbidities and mortalities. Therefore, healthcare providers in general, and physicians in particular, have an essential role in promoting smoking cessation in their patient encounters. The findings of the current study revealed that despite the knowledge of the participating Arab male physicians regarding the consequences of smoking and their professional obligation to encourage smoking cessation, many still smoke and are inadequately engaged in advising patients to quit smoking. These findings are supported in the literature [9, 10].

Characteristics of the Arab physicians, their previous experience, gender, social factors like ethnicity, and the Arab community culture, all influence their smoking-related behaviors, and normative and control beliefs. The Theory of Planned Behavior [26] posits that behavior is determined by one's intention to perform the behavior, and that intention is determined by three factors: attitudes, subjective norms, and perceived control. Normative beliefs refer to one's beliefs about whether important referents (e.g. friends, family) approve or disapprove of the behavior. These normative beliefs shape the subjective norm—one's perception that social pressure or expectations exist to perform or not perform the behavior.

In the case of smoking, if Arab physicians believe that their friends, parents, spouse etc. approve smoking (as normative belief), they will feel social pressure to smoke (subjective norm). Control beliefs refer to one's beliefs about the presence or absence of factors that facilitate or impede behavior. Control beliefs shape perceived behavioral control—one's perceived ease or difficulty in performing the behavior. If physicians believe that it would be hard to resist smoking when they are staying with their relatives or friends (control belief), their perceived ability to refrain from smoking in tempting situations (perceived control) decreases.

Our study revealed that smoking Arab male physicians have a desire for workshops on quitting smoking and would like them to be conducted during their working hours. We believe this is an important recommendation.

Smoking also was perceived by Arab males as an adaptive and normative way to deal with stress that emanates from academic studies and exams, economic strains, and workplace demands. Additionally, these smoking physicians experienced a stressful conflict between being physicians who are expected not to smoke while at the same time grappling with their own smoking addiction. In line with the Theory of Planned Behavior, being able to cope with stress will strengthen the physician's control and enable them to overcome their addiction. Accordingly, we recommend that health care managers provide workshops which might use techniques such as mindfulness or peer support groups to help physicians cope with stress.

A recent study amongst Palestinian physicians from Gaza found lower levels of knowledge about smoking addiction and management [31]. Physicians in that study reported that when they took the history of their patients on admission, they noted the patient's smoking behavior; and most referred patients who smoked to their family physician for further guidance about smoking cessation. Most of the participants in our study lacked sufficient knowledge and skills about how to quit smoking and to how to counsel their patients towards smoking cessation. Several studies as well as the TPB showed that physicians who have higher levels of knowledge and positive attitudes towards quitting smoking have more smoking cessation counseling encounters with their patients [26, 32–34]. This reinforces our recommendation that workplaces and organizations, such as health maintenance organizations, should develop and implement specific programs that will enhance the ability of smoking physicians to quit and equip all physicians to provide appropriate counseling and support to smoking patients.

### Limitations

The sample size in this study is small, but that is a common characteristic of qualitative studies. According to grounded theory [35], the criterion that determines the sample size of a qualitative study is the level of saturation measured by the recurrence of insights in interviews. Although our study sample was relatively small, it reached saturation levels. But the sample size of our study is not just small. It also reflects a low participation rate (25 out of 200 smoking Arab male physicians who were approached). Low response rates among physicians have been identified in other studies as well, and the reasons for this have included heavy workloads and a lack of time [36]. Lack of time for a one-hour interview was definitely a factor in our study. Nevertheless, when the participation rate is low, it always raises the question of whether the sample is representative of the population. Although it would take a quantitative study to answer that question, we feel that the responses given by our sample intuitively make sense, i.e., have face validity, and lead to important recommendations.

## Conclusions

The findings of the current study shed light on the complexity of the phenomenon of smoking behavior among professionals.

Summary of major points of the study:

- Arab populations have high smoking rates and smoking is considered an accepted cultural norm for some specific groups.
- Physicians face pressure both from their advanced studies requirements and other life stressors.
- Smoking has been a useful and acceptable way of coping with stress among Arab physicians.
- Arab physicians are aware of their smoking addiction, which is rooted in their cultural norms and serves as a way of coping with stress.
- There is no consensus among Arab male physicians regarding whether it is a professional responsibility for them to act as positive role models for patients and the public by not smoking.
- Physicians lack sufficient knowledge regarding smoking cessation both for themselves and for their patients.

We recommend supporting physicians in coping with pressures by establishing stress management programs and stress. We also recommend developing smoking cessation programs for physicians, especially for Arab male physicians among whom the prevalence of smoking is high. We further recommend that programs be developed and implemented to provide all physicians with the knowledge and skills to counsel their smoking patients effectively. When physicians not only understand but take on the obligation to be non-smoking role models to their patients they will enhance the image of the medical profession and, most importantly, improve the health of the public.

## Data Availability

The datasets used and analyzed during the current study are available from the corresponding author on reasonable request.

## Appendix

### Interview guide—Study on Factors Contributing to Smoking among Arab Physicians in Israel

Activate the recording application on your mobile device.

I am ––-, a research assistant in the study. We are conducting a research study on smoking among Arab physicians in Israel and would like to invite you to participate. We would like to thank you for agreeing to participate. The purpose of this research is to deeply understand the factors related to smoking among Arab physicians in Israel. Additionally, the study aims to examine the perceptions and attitudes of physicians regarding smoking and smoking cessation in general, as well professionals.

1. Background Information: Demographic Data—Please provide information on gender (recorded without asking), age, place of residence (village/city/mixed urban area), marital status, and number of children (if any). Occupation—Specify whether you have a specialization, your workplace (hospital/community/integrated), and the number of years you have been working as a physician. Smoking Habits—Include the number of years of smoking, the type of tobacco used (cigarettes, hookah, pipe, other), and the places where you typically smoke (designated smoking areas, public places, anywhere, other).
2. Attitudes toward smoking: What is your general opinion on smoking? What do you think about smoking among physicians? In your opinion, what are the reasons that lead physicians to continue smoking? Do you smoke in public places? How does being a physician affect your decision to smoke in public? What helped you quit smoking, if applicable? What do you know about smoking cessation methods and their effectiveness? What do you think should be a physician’s role regarding smoking? How do you react when a patient tells you that he smokes and finds it difficult to quit? What factors, whether personal, patient-related, or workplace-related, have facilitated smoking cessation for you?
3. Additional Comments: Is there anything else you would like to add?

If not, thank you very much for your cooperation. You may inquire whether the participant would like to receive a copy of the results, and if so, record their email for future communication.

## Abbreviations

COREQ: Consolidated criteria for reporting qualitative studies
PCP: Primary care physician
